# The *C. elegans* Opa1 Homologue EAT-3 Is Essential for Resistance to Free Radicals

**DOI:** 10.1371/journal.pgen.1000022

**Published:** 2008-02-29

**Authors:** Takayuki Kanazawa, Mauro D. Zappaterra, Ayako Hasegawa, Ashley P. Wright, Erin D. Newman-Smith, Karolyn F. Buttle, Kent McDonald, Carmen A. Mannella, Alexander M. van der Bliek

**Affiliations:** 1 Department of Biological Chemistry, David Geffen School of Medicine, University of California Los Angeles, Los Angeles, California, United States of America; 2 Resource for the Visualization of Biological Complexity, Wadsworth Center, Albany, New York, United States of America; 3 Electron Microscope Laboratory, University of California Berkeley, Berkeley, California, United States of America; Stanford University, United States of America

## Abstract

The *C. elegans eat-3* gene encodes a mitochondrial dynamin family member homologous to Opa1 in humans and Mgm1 in yeast. We find that mutations in the *C. elegans eat-3* locus cause mitochondria to fragment in agreement with the mutant phenotypes observed in yeast and mammalian cells. Electron microscopy shows that the matrices of fragmented mitochondria in *eat-3* mutants are divided by inner membrane septae, suggestive of a specific defect in fusion of the mitochondrial inner membrane. In addition, we find that *C. elegans eat-3* mutant animals are smaller, grow slower, and have smaller broodsizes than *C. elegans* mutants with defects in other mitochondrial fission and fusion proteins. Although mammalian Opa1 is antiapoptotic, mutations in the canonical *C. elegans* cell death genes *ced-3* and *ced-4* do not suppress the slow growth and small broodsize phenotypes of *eat-3* mutants. Instead, the phenotypes of *eat-3* mutants are consistent with defects in oxidative phosphorylation. Moreover, *eat-3* mutants are hypersensitive to paraquat, which promotes damage by free radicals, and they are sensitive to loss of the mitochondrial superoxide dismutase *sod-2*. We conclude that free radicals contribute to the pathology of *C. elegans eat-3* mutants.

## Author Summary

Dominant Optic Atrophy is a progressive eye disease caused by degeneration of retinal ganglion cells. The most prevalent form of DOA is caused by mutations in the Opa1 protein. This protein is required for fusion between mitochondria, it has an anti-apoptotic function, and it is required for mitochondrial DNA segregation. It has, nevertheless, been difficult to understand why mutations in Opa1 specifically affect retinal ganglion cells. We used rhe nematode *C. elegans* as a model to study the underlying causes of Opa1 pathologies. *C. elegans* Opa1 is encoded by the *eat-3* gene. Mutants are sluggish, grow slowly, remain small, and have small broodsizes. These phenotypes are not suppressed by mutations in cell death genes, suggesting that apoptosis does not contribute to *eat-3* pathogenesis. Instead, *eat-3* mutants are hypersensitive to paraquat, which promotes damage by free radicals, and they are sensitive to loss of the mitochondrial superoxide dismutase *sod-2*, which is needed to eliminate free radicals from the mitochondrial matrix. Moreover, *eat-3* mutants overexpress SOD-2, most likely compensating for increased free radical production. These results show that *C. elegans* EAT-3 is important for resistance to free radicals and they raise the possibility that free radicals contribute to DOA in humans.

## Introduction

Dominant optic atrophy (DOA) is one of the leading causes of inherited blindness. DOA is a progressive eye disease caused by degeneration of the retinal ganglion cell layer with ascending atrophy of the optic nerve [1]. The most prevalent form of DOA is caused by heterozygous mutations in the nuclear encoded, but mitochondrially targeted, Opa1 protein [2],[3]. Opa1 is a member of the dynamin family of proteins. This family consists of several large GTP binding proteins with diverse cellular functions. The archetypal dynamin is required for endocytosis [4],[5], but two other dynamin-related proteins, Drp1 and Mitofusins in mammals, act along with Opa1 to control mitochondrial fission and fusion. Mitochondrial fission and fusion are dynamic processes required for the replenishment of mitochondria, for example in long neuronal projections and during cell growth and division. Mitochondrial fission facilitates the redistribution of mitochondria in response to local changes in the demand for ATP, while mitochondrial fusion is needed to exchange mtDNA and other components that may become damaged over time [6],[7]. The rates of fission and fusion vary depending on cell type and environmental cues, but these rates are usually balanced. This balance is controlled by the opposing actions of the different dynamin family members on or in mitochondria.

The three dynamin-related proteins that affect mitochondria have different topologies and play different roles in fission and fusion. Mammalian Drp1 and the homologous proteins in C. elegans and yeast are cytosolic proteins that are required for mitochondrial division [8]–[10]. These proteins wrap around constricted parts of mitochondria where they control a late stage of mitochondrial outer membrane division [8],[11]. Mutations in Drp1 homologues give rise to a highly interconnected mesh of mitochondria [8]–[10]. Fusion between mitochondrial outer membranes is mediated by a different set of dynamin family members [12]. These proteins are called Mitofusins in mammals and Fzo1 in yeast and Drosophila. They have two transmembrane segments that anchor the proteins in the mitochondrial outer membrane. There are two Mitofusins in mammals (Mfn1 and Mfn2), which are often coexpressed but are not redundant [13]. Mutations in Mfn2 cause peripheral neuropathy in Charcot Marie Tooth (CMT) disease [14]. Mutations in Fzo1 and Mitofusins give rise to fragmented mitochondria [12],[15],[16], but this fragmentation can be suppressed by mutations in Drp1 homologues in yeast and mammalian cells.

Evidence for the role of Opa1 in fusion between mitochondrial inner membranes initially came from studies of the yeast homologue of Opa1, which is called Mgm1. The mitochondria of yeast Mgm1 mutants are fragmented, they form aggregates and they lose their mtDNA [17]–[19]. Conditional mutations show that the loss of mtDNA is preceded by the changes in mitochondrial morphology, indicating that loss of mtDNA is a secondary defect [18]. The mitochondrial fragments in Mgm1 mutants are converted into a closed network of mitochondria by additional mutations in mitochondrial fission proteins, suggesting that Mgm1 is a mitochondrial fusion protein [20]–[23]. This role was substantiated by experiments in which two yeast cells with differently labeled mitochondria are allowed to fuse. The mitochondria of Mgm1 mutant cells do not mix the two labels showing that they are unable to fuse [24]. A direct role in mitochondrial fusion was then shown with *in vitro* reconstitution experiments using mitochondria isolated from yeast Mgm1 mutants [25].

Biochemical analysis shows that yeast Mgm1 and mammalian Opa1 are localized to the mitochondrial intermembrane space [19],[24],[26],[27]. The mitochondrial leader sequences of Mgm1 and Opa1 are cleaved upon import into mitochondria. In yeast, roughly half of the protein is further processed by a rhomboid protease [28]–[32]. A homologue of this protease, called PARL, exists in mammals, but cleavage in higher eukaryotes may require other proteases [33]. Immuno-electron microscopy of mammalian cells shows the bulk of Opa1 protein distributed throughout cristae with only a small portion localized to the boundary space between mitochondrial inner and outer membranes [27].

The importance of Opa1 for housekeeping functions, such as mitochondrial fusion and redistribution of mtDNA, is apparent from these cell biological studies. It has, nevertheless, been difficult to establish the exact sequence of events leading to retinal ganglion cell death in DOA, even with the mouse models that have recently become available [34],[35]. The effects on retinal ganglion cells are restricted both in time and place and they occur with the mild loss of Opa1 function that results from haploinsufficiency of the Opa1 gene [36]. In contrast, cultured mammalian cells transfected with Opa1 siRNA typically show the stronger effects that are associated with complete loss of Opa1 function. Late time points after transfection with Opa1 siRNA show mitochondria that are reduced to small dispersed fragments [27],[37],[38], while early time points show that this fragmentation is preceded by internal rearrangements of the mitochondrial inner membrane [27]. At these times the mitochondria swell and stretch forming localized constrictions, similar to the changes in mitochondrial morphology that are observed during early stages of apoptosis [39]. Transfection with Opa1 siRNA also increases susceptibility to apoptosis by promoting cytochrome c release [40]. Increased susceptibility to apoptosis, exacerbated by photo-damage, was therefore proposed as a possible cause of retinal ganglion cell death in patients with DOA [41]. However, alternatives, such as the effects of reduced levels of ATP, are also considered as possible causes of DOA [42].

Here we show that the previously described *C. elegans eat-3(ad426)* strain [43] has a mutation in the D2013.5 gene, which encodes the ortholog of yeast Mgm1 and mammalian Opa1. The *ad426* mutation leads to fragmented mitochondria similar to those cause by mutations in Opa1 and Mgm1. Electron microscopy shows that *eat-3(ad426)* mitochondria have disorganized inner membranes and a large number of inner membrane septae. We also find that *eat-3(ad426)* growth defects are attributable to impaired oxidative phosphorylation and increased damage from free radicals within mitochondria.

## Results

### C. elegans EAT-3 Is an Orthologue of Yeast Mgm1 and Mammalian Opa1

**Figure 1:**
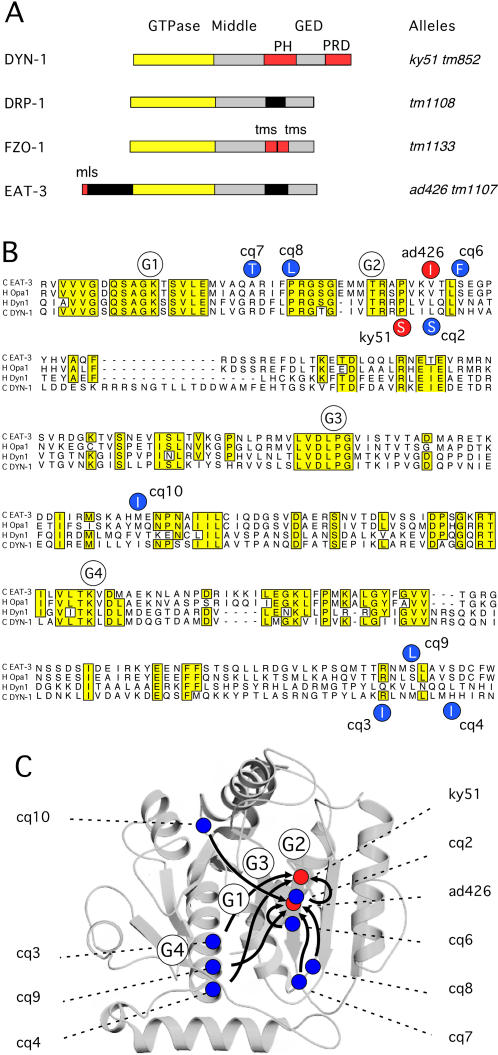
Mutations in *C. elegans eat-3* and *dyn-1* Mutants. (A) Dynamin family members in *C. elegans*. DYN-1 is required for scission of vesicles from the plasma membrane. DRP-1 is required for scission of mitochondrial outer membranes. FZO-1 is required for fusion of mitochondrial outer membranes. EAT-3 is required for fusion of mitochondrial inner membranes. The GTPase, Middle, and GTPase Effector (GED) domains are shared between dynamin family members. In addition, DYN-1 has a pleckstrin homology (PH) domain and a proline rich domain (PRD), FZO-1 has two transmembrane segments that anchor the protein in the mitochondrial outer membrane, and EAT-3 has a mitochondrial leader sequence (mls) that targets the protein to the mitochondrial intermembrane space. Some key alleles are shown on the right. (B) Sequence alignment of the GTPase domains of *C. elegans* EAT-3 (D2031.5), human Opa1, human Dyn1, and *C. elegans* DYN-1. The GTP binding consensus sequences (G1-4) are indicated with white circles. The primary mutations in *eat-3(ad426)* and *dyn-1(ky51)* alleles are shown in the red circles. Secondary mutations in the intragenic revertants are shown in the blue circles. The *dyn-1(ky51)* revertants *cq2, cq3,* and *cq4* are shown below the sequences and the *eat-3(ad426)* revertants *cq6, cq7, cq8, cq9,* and *cq10* are shown above the sequences. (C) The positions of the GTP binding motifs (open circles labeled G1-4), the positions of primary mutations (red circles), and the positions of secondary mutations (blue circles) superimposed on the structure of the rat Dyn1 GTPase domain [47]. The arrows point to the primary mutation suppressed by each secondary mutation.

BLAST homology searches show that *C. elegans* has a single homologue of yeast Mgm1 and mammalian Opa1. This protein is encoded by the D2013.5 gene. It has a predicted molecular weight of 106.8 kDa and 46% amino acid identity to human Opa1. Similar to yeast Mgm1 and mammalian Opa1, this *C. elegans* protein has a putative mitochondrial targeting sequence followed by domains that are typical of dynamin family members: a conserved GTPase domain, a middle domain and a GED or assembly domain [44] (Figure 1A). Pilot experiments with D2013.5 RNAi yielded worms that grew slowly, remained small and had small numbers of progeny. These phenotypes led us to investigate the *eat-3* mutant, which was previously identified in a screen for mutations that cause abnormal or defective eating in *C. elegans* [43]. The D2013.5 gene is very close to the *eat-3* locus (within 0.2 map units) and the overall appearance of D2013.5 RNAi animals is similar to that of *eat-3* animals.

Upon sequencing the D2013.5 gene from *eat-3(ad426)* animals, we found a single point mutation, changing a valine at position 328 to an isoleucine (Figure 1B). Although this is a surprisingly conservative change, there are other examples where such a change has a dramatic effect on protein function [45]. The affected residue is just downstream of the G2 threonine in the effector binding loop of the dynamin-like GTPase, where it may disrupt the GTPase cycle. A *C. elegans* dynamin mutant, *dyn-1(ky51)*, has a mutation that is also very close to the G2 threonine [46]. Surprisingly, this dynamin mutation can be suppressed by a second mutation at the same position as that mutated in *eat-3(ad426)*, which further demonstrates the importance of this particular residue (Figure 1B).

To verify that *eat-3(ad426)* is indeed a D2013.5 mutant, we injected this strain with a wildtype D2013.5 cDNA under control of the D2013.5 gene promoter. The number of progeny reaching the L4 larval stage increased from 10 per uninjected *eat-3* animal (SD = 8, n = 28) to 30 per transgenic animal (SD = 22, n = 26), showing that a wildtype D2013.5 construct partially rescues the *eat-3* mutant. Partial rescue is common for *C. elegans* genes with a maternal effect, since transgenes are often poorly expressed in the germline. We obtained further evidence that D2013.5 encodes the *eat-3* locus with a second allele, named *tm1107*. The *tm1107* allele is most likely a null, since it has a 419 bp deletion that causes a frameshift at position 329 and thus eliminates two thirds of the D2013.5 protein. The absence of EAT-3 protein in *tm1107* animals, but not in ad426 animals, was confirmed by Western blot analysis using an antibody raised against the *C. elegans* EAT-3 protein (Figure S1). Homozygous *eat-3(tm1107*) animals survive but they have fragmented mitochondria, a decrease in broodsize, sluggishness and slow growth phenotypes, similar to the phenotypes of *eat-3(ad426*) animals. More importantly, *tm1107* fails to complement *eat-3(ad426*), indicating that *ad426* and *tm1107* are both alleles of *eat-3* and that the phenotypes are due to mutations in the D2013.5 gene (data not shown). The *C. elegans* D2013.5 locus is henceforth called *eat-3*.

Additional alleles of *eat-3* were isolated in an F2 screen for suppressors of *eat-3(ad426)* mutant phenotypes. The progeny of 36,000 F1 animals were screened for restored growth rate, size and fecundity. This screen yielded seven new mutants with restored growth rates. Five of these mutants have second site mutations in the *eat-3* gene (*cq6-cq10*), while two mutants have mutations that lead to premature stops in the *drp-1* gene (*cq5* and *cq11*). The new mutations in the *eat-3* locus all cause substitutions in the GTPase domain (Figure 1B). A similar screen with the *dyn-1(ky51)* also yielded a series of substitutions in the GTPase domain (Figure 1B). When the new mutations are mapped onto the crystal structure of the dynamin GTPase domain [47], they reveal a striking pattern of convergence on the G2 motif of the GTPase domain (Figure 1C). It seems likely that they restore the ability of the G2 threonine to interact properly with GTP or make the conformational changes that occur during GTP hydrolysis.

### Fragmented Mitochondria Caused by Mutant eat-3 or Loss of EAT-3 Protein

To investigate how *eat-3* affects mitochondria, we focused on mitochondrial morphology in *C. elegans* body wall muscles. Mitochondria were detected with mitochondrial matrix markers, consisting of an N-terminal mitochondrial leader sequence fused to GFP, cyan fluorescent protein (CFP) or yellow fluorescent protein (YFP), and mitochondrial outer membrane markers, consisting of a resident outer membrane protein (TOM70) fused to GFP, CFP or YFP [8]. The functions of the EAT-3 protein were disrupted by expressing dominant negative mutant proteins or antisense cDNA, which effectively causes localized RNAi, under control of the muscle specific *myo-3* promoter. The dominant negative mutations that we used here are T322A, which disrupts the G2 motif of the GTPase domain, and K300A, which is analogous to the K44A mutation in the G1 motif of dynamin [5].

**Figure 2:**
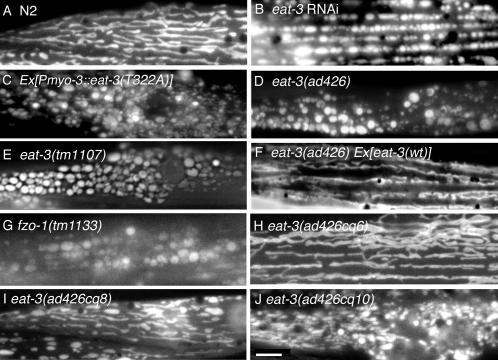
Fragmented Mitochondria Caused by RNAi or Mutations in *eat-3.* (A) Regular tubular array of mitochondria in muscle cells of a wildtype worm. (B) Fragmented mitochondria in muscle cells subjected to *eat-3* RNAi generated with a P*myo-3:: antisense-eat-3* construct. (C) Fragmented mitochondria in muscle cells of worms with a P*myo-3:: eat-3(T322A)* construct. (D) Fragmented mitochondria in muscle cells *eat-3(ad426)* worms. (E) Fragmented mitochondria in the muscle cells of *eat-3(tm1107)* worms. (F) Tubular mitochondria in the muscle cells of *eat-3(ad426)* worms that were rescued by a construct containing wildtype *eat-3* cDNA under control of the *eat-3* gene promoter (Ex[eat-3(wt)]). (G) Mitochondria in the *fzo-1(tm1133)* deletion strain. (H-J) Mitochondria in the *eat-3(ad426)* revertants *cq6*, *cq8,* and *cq10*. Mitochondria were detected with matrix GFP driven by the muscle specific myo-3 promoter. The bar indicates 5 µm.

Both dominant negative mutations and the loss of function induced by antisense cDNA cause mitochondria to fragment into a large number of small pieces (Figure 2B and 2C; 83% of cells were affected, n = 200). Labeling with a mitochondrial outer membrane marker shows that the mitochondrial fragments truly are detached (not shown). The exact size and distribution of mitochondrial fragments varied between the different treatments, but it was not evident that these phenotypes represent different levels of severity (Figure 2B–2C). Mitochondrial fragmentation is also observed in *eat-3(ad426)* and in *eat-3(tm1107)* animals and this phenotype is reversed in transgenic animals expressing wildtype *eat-3* cDNA under control of the *myo-3* promoter (Figure 2D–2F).

Similar mitochondrial fragmentation is observed in muscle cells of *fzo-1(tm1133)* mutants (Figure 2G), which have a mutation in the *C. elegans* homologue of *Drosophila* and yeast Fzo1 and mammalian Mitofusins, proteins required for fusion of the mitochondrial outer membrane [12],[13],[15],[16],[48],[49]. The mitochondria of *eat-3* and *fzo-1* mutants are similarly fragmented consistent with their roles in mitochondrial fusion. However, the gross anatomical defects (size, growth rate and broodsize) are less severe in *fzo-1(tm1133)* mutants and *fzo-1* RNAi animals than in *eat-3* mutants (data not shown), even though *fzo-1(tm1133)* is also a null allele (it has a chromosomal deletion that truncates the protein after 65 amino acids). These results suggest that there might be functional differences in the ways that EAT-3 and FZO-1 proteins affect the gross anatomy of *C. elegans*.

In contrast, intragenic revertants of *eat-3(ad426)* show a range of mitochondrial morphology defects (Figure 2H–2J); the mitochondria are still fragmented in *eat-3(ad426cq10)*, they are partially restored to their filamentous morphology in *eat-3(ad426cq8)* and completely restored in *eat-3(ad426cq6)* commensurate with the suppression of gross anatomical defects. We conclude that mutations in *fzo-1* and *eat-3* both cause mitochondrial fragmentation, but their effects on size, growth rate and broodsize are different.

### Ultrastructural Analysis of Wildtype and eat-3 Mutant Mitochondria

**Figure 3:**
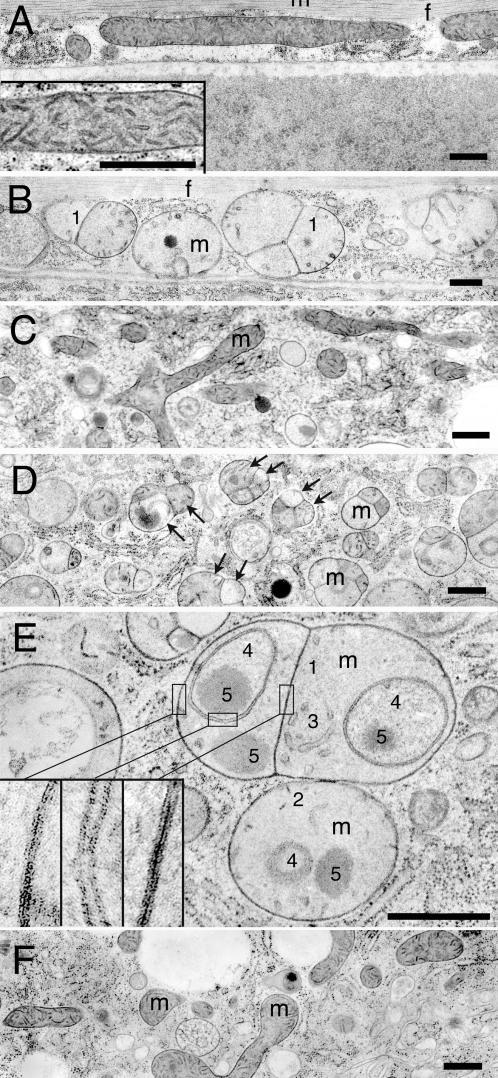
Aberrant Internal Structures in *eat-3(ad426)* Mitochondria. (A) Longitudinal section of a body wall muscle in a wildtype worm. This section shows one long mitochondrion (m) and myofibrils (f). The insert is an enlargement of a muscle cell mitochondrion. (B) Longitudinal section of a body wall muscle in an *eat-3(ad426)* worm. (C) Section of an intestinal cell in a wildtype worm. The mitochondria (m) are randomly oriented and therefore sectioned at many different angles. (D) Section of an intestinal cell in an *eat-3(ad426)* worm. The pairs of arrows indicate matrix compartments with different cristae morphologies enclosed by a single outer membrane. (E) Enlarged portion of an intestinal cell showing mitochondria (m) with more inner membrane aberrations. Aberrant structures indicated with numbers: 1. Inner membrane septae. 2. Short tubular cristae. 3. Long invaginations of inner membrane. 4. Novel compartments within the mitochondrial matrix enclosed by a double membrane. 5. Electron dense inclusions in the mitochondrial matrix. The inserts show further enlargements of three different places with double membranes. (F) Section of an intestinal cell in an *eat-3(ad426)* worm rescued by microinjection of a wildtype *eat-3* transgene. Bars are 0.5 µm.

To further investigate how mitochondria are affected, we conducted electron microscopic analysis of wildtype and *eat-3(ad426)* worms. Figure 3A and 3C show longitudinal sections of wildtype worms. The mitochondria in muscle cells are long, while mitochondria in other cell types, such as intestinal cells, appear to be short or round, because they are randomly oriented with respect to the plane of sectioning. The mitochondria contain many short pairs of membrane segments that criss-cross the mitochondrial matrix (Figure 3A, insert). These segments are likely to be oblique sections of randomly oriented cristae. Their morphology suggests that *C. elegans* mitochondria contain tightly packed tubular cristae.

In contrast, *eat-3* mitochondria are almost all round (Figure 3B, 3D, and 3E) and often further divided by inner membrane septae (“1” in Figure 3E). The number of mitochondria transected by inner membrane septae, as detected in thin sections, is less than 0.5% in wildtype animals (n = 220) and 63% in *eat-3(ad426)* animals (n = 221). The frequency of internal septae in *eat-3* animals is likely to be even higher, because the thin sections will have missed septae outside of the plane of sectioning. We conclude that the majority of *eat-3(ad426)* mitochondria are divided by internal membrane septae. In contrast, wildtype mitochondria are rarely if ever further divided by septae.

The mitochondria of *eat-3*(ad426) animals often have shorter and reduced numbers of cristae. These cristae typically project no more than 100 nm into the matrix (“2” in Figure 3E), while cristae in wildtype mitochondria are more densely packed and appear to traverse the width of mitochondria. To quantify the difference between *eat-3* and wildtype cristae, we traced the lengths of mitochondrial membranes detected in thin sections. The *eat-3* mitochondria had on average 1.21 µm total cristae length (n = 20, SD = 0.78), compared with 7.34 µm in wildtype mitochondria (n = 16, SD = 3.80). The length of inner boundary membranes is also decreased: 2.62 µm per *eat-3* mitochondrion (n = 20, SD = 0.91), compared with 5.38 µm per wildtype mitochondrion (n = 16, SD = 2.64). There is, however, still a 66.2% decrease of total cristae length when normalized with the lengths of inner boundary membranes or a 70.3% decrease when normalized with the surface area of the mitochondrial section. We conclude that most *eat-3* mitochondria have fewer cristae than wildtype mitochondria.

Some mutant mitochondria have long inner membrane invaginations, which could in principle be enlarged cristae, but are more likely membrane folds resulting from a surplus of inner membrane (“3” in Figure 3E). It is, however, not clear from the EM sections whether these membrane folds are attached to the rim. In addition, many *eat-3* mitochondria have internal curved or ring-shaped structures formed by two concentric membranes enclosing a matrix-like material (“4” in Figure 3E). These membrane inclusions are *eat-3-*specific, since they were not observed in wildtype animals. The matrices of *eat-3* mitochondria also contain electron-dense inclusions (“5” in Figure 3E), but these are not specific for the *eat-3* mutant, since wildtype mitochondria contain similar (albeit smaller) inclusions. Given their internal location, all of these membrane inclusions are likely to be derived from the inner membrane.

**Figure 4:**
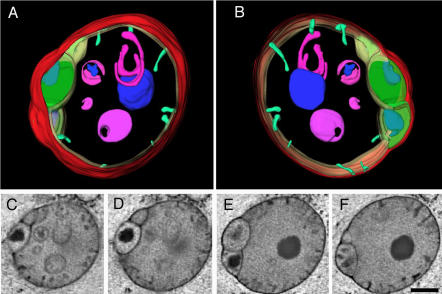
Tomographic Reconstruction of an *eat-3(ad426)* Mitochondrion. (A,B) Front and rear views of an *eat-3* mitochondrion reconstructed by electron tomography. Outer membrane is shown in red. Inner membrane is shown in green. Bubbles on the side of the mitochondrion, which are also shown in green, are matrix compartments separated inner membrane septae. Invaginations of the inner membrane, which are likely to be short tubular cristae, are shown in light green. Internalized membrane-bound compartments are shown in magenta. Large clusters of electron dense material without membrane are shown in blue. (C–F) Tomographic sections used to make the three-dimensional renderings in (A) and (B). The bar is 0.5 µm.

To determine whether the various membrane inclusions observed in thin sections are connected outside of the plane of view, we made three-dimensional reconstructions of mitochondria using electron tomography. In this technique, a thick section is viewed at different angles and the imaging data is used to reconstruct a three dimensional model. Images of an *eat-3* mitochondrion are shown in Figure 4. This mitochondrion has several matrix “bubbles”, divided from the rest of the matrix by septae of inner membrane. These bubbles are sealed off, indicating that the septae observed in the thin sections reflect completed inner membrane divisions. The three-dimensional reconstructions of *eat-3* mitochondria also show that some of the membrane inclusions that appear free floating in the matrix are indeed physically separated from the inner membrane (Figure 4). This separation suggests severing of membranes within the mitochondrial matrix by an as yet unknown mechanism. Similar free-floating structures were previously observed in a mitochondrial myopathy of unknown etiology in humans [50] and in mitochondria of apoptotic cells [51].

### Inhibition of Growth by eat-3 RNAi

To investigate the role of *eat-3* in whole worms we first determined the expression pattern of the *eat-3* gene with transgenic animals that carry an extrachromosomal array with the *eat-3* gene promoter fused to green fluorescent protein (GFP) and β-galactosidase coding sequences. This pattern was similar to that of *C. elegans drp-1* [8] with high levels in intestinal cells, in muscle cells and in neurons and low levels in other cell types (data not shown). Cell types with high levels of expression may be metabolically more active than other cell types, but basal levels of this protein are most likely required in all cells.

We then conducted experiments to assess the effects of *eat-3* loss of function on the growth and brood size of worms. Worms injected with *eat-3* dsRNA give viable progeny but their brood size is reduced (90 viable eggs per worm, SD = 14, n = 20, compared with 270 for wildtype, SD = 10, n = 20). The F1 worms remain small, are sluggish and develop slowly. Similar effects were observed with chromosomal mutations in the *eat-3* gene. In an experiment with *ad426* and *tm1107* alleles, the averages were 302 for wildtype (SE = 8.2, n = 7), 51 for *eat-3(ad426)* (SE = 8.4, n = 7) and 50 for *eat-3(tm1107)* (SE = 10, n = 7). In an experiment with intragenic revertants of *eat-3(ad426)*, the averages were 75 for *eat-3(ad426cq6)* (SE = 48, n = 6), 190 for *eat-3(ad426cq7)* (SE = 14, n = 6) and 65 for *eat-3(ad426cq8)* (SE = 27, n = 6). These numbers are variable, as one might expect from different allele strengths, but they are all reduced when compared with wild type animals.

**Figure 5:**
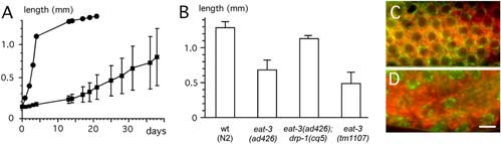
Slow Growth and Disrupted Gonads Caused by *eat-3* RNAi. (A) Growth curve comparing wildtype worms to the progeny of worms injected with *eat-3* RNAi. The plots show the average lengths obtained with 5 wildtype worms (closed circles) and 20 *eat-3* RNAi progeny (closed squares) with standard deviations. (B) Chromosomal *eat-3* mutants are also smaller than wild type, but this defect can be restored by a mutation in *drp-1*. The lengths of *eat-3(ad426)*, *eat-(ad426); drp-1(cq5)* and *eat-3(tm1107)* are compared with the lengths of wild type (N2) animals four days after hatching. The lengths are averages of 12, 13, 5, and 16 animals respectively with standard deviations. (C) Close-up of a gonad arm from a wild type worm. Mitochondria were stained with rhodamine 123 (red) and nuclear DNA was stained with Hoechst (green). The nuclei form an orderly pattern near the surface of the gonad. These nuclei are always surrounded by mitochondria. (D) Gonad of a worm injected with *eat-3* RNAi. The gonads are dissected two days after injection with dsRNA. The mitochondria appear more dispersed than in wildtype but are not notably less abundant. Instead, there is a paucity of nuclei consistent with the reduced brood size of *eat-3* RNAi animals. The bar is 5 µm.

Growth was quantified by measuring the lengths of progeny from RNAi injected worms (Figure 5A). Progeny from worms injected with *eat-3* dsRNA were on average only 0.15 mm in length at four days after hatching (SD = 0.02, n = 20), whereas wild type animals were 1 mm in length (SD = 0.04, n = 5). Even after three weeks, the eat-3 RNAi worms rarely reach 0.5 mm, consistent with a previous study showing that the *eat-3(ad426)* mutant also remains small [52]. Similar effects on length were observed with chromosomal mutations in the *eat-3* gene (Figure 5B). Worms with *eat-3* deficiencies live longer than wildtype animals (33 days for RNAi worms, versus 20 days for untreated worms, and as shown previously with *eat-3(ad426)* animals [53]), but it also takes them longer to reach adulthood (10 days for *eat-3* RNAi progeny whereas wildtype animals take 2 days). It would thus appear that developmental decisions are normal, but the rate of development is greatly reduced as one might expect from a general decrease in metabolic activity.

To see how mitochondria in the gonads of *eat-3* RNAi animals are affected, we stained the gonads of injected worms with Rhodamine 123, as was previously done with *C. elegans drp-1* RNAi animals [8]. We find that the mitochondria are more dispersed, but do not appear to be less abundant than in untreated gonads (Figure 5C–5D). The effect of *eat-3* RNAi on mitochondria is, however, much less dramatic than that of *drp-1* RNAi, which causes mitochondria to form large aggregates [8]. However, Hoechst staining shows that there is a paucity of nuclei when compared with wildtype (Figure 5C–5D). This paucity suggests reduced numbers of mitotic divisions at the tips of the gonads, which would lead to the production of fewer oocytes in agreement with the low brood sizes of *eat-3* mutant and RNAi treated animals.

### Genetic Interactions between C. elegans eat-3, drp-1, and fzo-1

**Figure 6:**
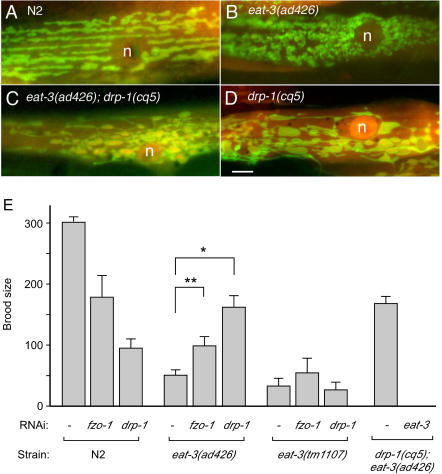
Suppression of *eat-3(ad426)* Phenotypes. (A) Mitochondria in a wildtype (N2) muscle cell with their normal tubular morphology. (B) Fragmented mitochondria in a muscle cells of the *eat-3(ad426)* mutant. (C) Mitochondria with highly connected outer membranes (green) but not connected matrix compartments (red) in a muscle cell of the *eat-3(ad426); drp-1(cq5)* double mutant. (D) Similarly connected mitochondrial outer membranes in a muscle cell of the *drp-1(cq5)* mutant after removal of the *eat-3(ad426)* mutation by backcrossing. The mitochondria in muscle cells were detected with the transmembrane segment of *C. elegans* Tom70 fused to YFP (shown in green) and the matrices are labeled with a mitochondrial leader sequence fused to CFP (red). Nuclei are marked with n. The bar indicates 5 µm. (E) Histograms showing rescue of the *eat-3(ad426)* broodsize defect by *drp-1* and *fzo-1* RNAi, but not rescue of the *eat-3(tm1107)* deletion allele by *drp-1* or *fzo-1* RNAi. Wildtype (N2) and mutant animals were grown on bacteria with the feeding RNAi plasmid pILL4440 without insert, with *fzo-1* cDNA or with *drp-1* cDNA. The bars on the right show that the brood size of the *eat-3(ad426)* mutant is also rescued by a chromosomal *drp-1* mutation (*drp-1(cq5)*). This rescue depends on residual *eat-3* function in the *ad426* allele, because it is eliminated by *eat-3* RNAi. The brood sizes were defined as the numbers of viable larvae that developed to the L4 stage. Error bars indicate SE. An unpaired Student's *t* test was used for statistical analysis. The single asterisk indicates P<0.0001 and the double asterisk indicates P<0.01 (n = 24 for *eat-3(ad426)* alone, n = 14 for the same with *fzo-1* RNAi and n = 7 for *drp-1* RNAi).

Two of the mutants that were isolated in our screen for suppressors of *eat-3(ad426)* have premature stop codons in the *drp-1* gene, showing that defects in mitochondrial fission suppress the defect in mitochondrial fusion caused by a mutation in *eat-3*. Similar genetic interactions were previously observed with mutations in the orthologous yeast genes [19],[21]. Since mitochondrial fission and fusion proteins not only act antagonistically on mitochondrial morphology, but also affect the viability of worms, we conducted additional experiments to further determine the extent of *eat-3* suppression by *drp-1* loss of function. First, we tested whether the fragmentation of mitochondria is reversed by the dominant negative mutant DRP-1(K40A), which blocks division of the mitochondrial outer membrane [8]. Constructs encoding P*myo-3::DRP-1(K40A)* and a mitochondrial outer membrane marker were injected into *eat-3(ad426)* worms or into wildtype worms along with the P*myo-3::antisense-eat-3* construct. DRP-1(K40A) gives rise to interconnected mitochondria, regardless of whether it is expressed in a wildtype background, with antisense *eat-3*, or in an *eat-3* mutant (100% of cells, n = 50, data not shown). The *drp-1(cq5)* allele, which was isolated as a suppressor of *eat-3(ad426)*, also causes hyperconnectivity of mitochondria in *eat-3(ad426)* animals, similar to the connectivity observed in the *drp-1(cq5)* single mutant (Figure 6A–6D). We conclude that a functioning mitochondrial division apparatus is required for the mitochondrial fragmentation induced by mutant *eat-3*.

To find out whether other abnormalities of the *eat-3(ad426)* mutant are reversed by a defect in mitochondrial division, we determined the brood-size of *eat-3(ad426)* mutants grown with or without *drp-1* RNAi. Our results show that *drp-1* RNAi significantly restores the brood-size of *eat-3(ad426)* mutants (Figure 6E). A chromosomal mutation in *drp-1* also restores the brood size as shown with *eat-3(ad426); drp1(cq5)* animals (Figure 6E). We conclude that defects in *C. elegans* DRP-1 and EAT-3 proteins compensate each other's physiological defects. Similar effects were observed in yeast, where the effects of mutations in the EAT-3 homologue Mgm1 are suppressed by mutations in the DRP-1 homologue Dnm1 [19],[21]. To our surprise, however, the brood size defect of the *eat-3(ad426)* allele was also partially suppressed by *fzo-1* RNAi, while the *eat-3(tm1107)* allele, which is most likely a null allele, was not suppressed by *drp-1* or *fzo-1* RNAi (Figure 6E), even though mitochondrial fragmentation in *eat-3(tm1107)* animals is reversed by *drp-1* RNAi (Figure S2). These results suggest that the *eat-3(ad426)* allele has some residual protein function that is masked by wildtype DRP-1 and FZO-1 proteins. In support of this residual activity, we find that *eat-3* RNAi reverses the restoration of brood size by the *drp-1* mutation in *eat-3(ad426); drp1(cq5)* animals (Figure 6E).

The suppressive effects of *drp-1* and *fzo-1* loss of function can be explained by the fact that they both act upstream of inner membrane fusion. Loss of *drp-1* prevents the formation of inner membrane fusion intermediates by introducing a fission defect that is epistatic to fusion defects, while loss of *fzo-1* does this by blocking outer membrane fusion, which also precedes inner membrane fusion. It seems likely that the inner membrane fusion intermediates, formed with wildtype *drp-1* and *fzo-1,* sequester mutant EAT-3 protein, while loss of *drp-1* or *fzo-1* function frees this protein for other essential functions within the mitochondrial intermembrane space.

### Failure of ced-3 and ced-4 Mutations To Suppress eat-3 Phenotypes

**Figure 7:**
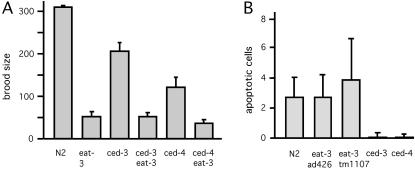
Lack of Evidence for Increased Cell Death in eat-3 Mutant Animals. (A) The broodsizes of *eat-3(ad426)* animals alone were not appreciably different from the broodsizes of the *ced-3(n717); eat-3(ad426) or ced-4(n1894); eat-3(ad426)* double mutants. Feeding RNAi for the other caspases (*csp-1, csp-2* and *csp-3*) also had no effect (data not shown). (B) Numbers of dying cells were counted in comma stage embryos of wildtype (N2), *eat-3(ad426)*, *eat-3(tm1107)*, *ced-3(n717)* and *ced-4(n1894*) animals. The numbers of dying cells were not significantly increased in the *eat-3* mutants when compared with wildtype. The numbers of dying cells in *ced-3(n717)* and *ced-4(n1894*) embryos were included to show that cell death is effectively blocked in these mutants. The bars indicate SE.

It is well-established that Opa1 has an anti-apoptotic function in mammalian cells [37],[40],[54],[55]. We therefore tested whether apoptosis contributes to the various *eat-3* phenotypes in *C. elegans* by making double mutants with *eat-3(ad426)* and *ced-3(n717)* or *ced-4(n1894)* mutations. The *ced-3* gene encodes a caspase and the *ced-4* gene encodes APAF-1. Mutations in either gene block programmed cell death in *C. elegans.* The effects on broodsize were determined by counting the numbers of progeny that survive to the L4 larval stage. The brood sizes were reduced to varying degrees in each of the single mutants, but the brood size defects of the *eat-3(ad426)* animals were not significantly affected by the additional mutations in *ced-3* and *ced-4* loci (Figure 7A). Although *ced-3* encodes the caspase that is utilized for all programmed cell death in *C. elegans* and inducible cell death in *C. elegans* gonads [56], there are three other caspases (*csp-1*, *csp-2* and *csp-3*) that might contribute to cell death under other circumstances. We tested these *csp* genes with feeding RNAi, but saw no effect on the brood size of *eat-3(ad426)* mutants. Some redundancy between the caspases remains possible, but redundancy does not apply to *ced-4*, since it encodes the single *C. elegans* homologue of APAF-1.

The *ced-4* gene is central to all caspase dependent cell death in *C. elegans*. The absence of an effect of *ced-4* mutations on the *eat-3* broodsize defect, as shown here (Figure 7A), is therefore a reliable indication that caspase dependent cell death does not contribute to the reduced broodsize of *eat-3* mutants. To verify that *eat-3* mutants show no increase in cell death, we counted the numbers of dying cells by looking for light-refractory cells with DIC microscopy in *eat-3(ad426)* and *eat-3(tm1107)* embryos at the comma stage. Those numbers were not significantly different from the numbers for wildtype embryos (Figure 7B). To verify that the *ced* mutants used here were effective, the numbers of dying cells were also counted in *ced-3(n717)* and *ced-4(n1894)* mutant embryos. As expected, these mutants show strongly reduced numbers of dying cells. We conclude that *ced-3* and *ced-4* dependent cell death does not contribute to the reduced brood size of *eat-3* animals. The *eat-3; ced-3* and *eat-3; ced-4* double mutants also grow slowly and remain small similar to the *eat-3* single mutants (data not shown), suggesting that cell death does not contribute to these other maladies.

### Paraquat Sensitivity of eat-3 Mutants

**Figure 8:**
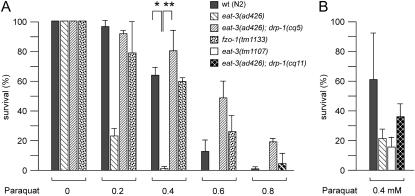
Paraquat Sensitivity of the *eat-3(ad426)* Mutant and Its Reversal by *drp-1(cq5).* (A) The histogram shows the percentages of worms that survive from the L1 larval stage to adulthood when grown on plates with increasing concentrations of paraquat. Fifty L1 larvae with the indicated genotypes were transferred to five fresh plates for each data point and monitored for five days. The entire experiment was done in triplicate, except for *drp-1(cq5); eat-3(ad426)*, which was done in duplicate. The bars show averages and SD for the experiments. An unpaired Student's *t* test was used for statistical analysis. The single asterisk indicates P<0.0001 and the double asterisk indicates P<0.0005 at 0.4 mM paraquat (n = 3). The N2 (wildtype) and *eat-3(ad426)* strains were tested two more times to more accurately determine the IC50 (see text). (B) A comparison between *eat-3(ad426)*, *eat-3(tm1107)* and *eat-3(ad426); drp-1(cq11)* at 0.4 mM paraquat. The histogram shows averages of three experiments with their standard deviations. The increased sensitivity of *eat-3(tm1107)* animals to paraquat confirms that the effect is due to loss of *eat-3* function, because it was observed with two independent alleles (*ad426* and *tm1107*). In this same manner, reversal of paraquat sensitivity by the *cq11* allele in *eat-3(ad426); drp-1(cq11)* animals confirm that the reversal is due to loss of *drp-1* function, because it was also observed with two independent alleles (*cq5* and *cq11*). The absolute numbers for N2 and *eat-3(ad426)* in panels A and B show minor differences because of variations between experiments, but the trends are the same.

The growth and brood size defects of *eat-3* mutants resemble those of *gas-1* and *mev-1* mutants, which have defects in Oxidative Phosphorylation complexes. Those mutants are also more susceptible to damage from free radicals, as shown by their sensitivity to paraquat, which produces superoxide radicals through a radical ion intermediate [57]. To test whether *eat-3* mutants are also sensitive to free radicals, we grew *eat-3(ad426)* animals with increasing concentrations of paraquat. We find that *eat-3(ad426)* animals are significantly more sensitive to paraquat than wildtype animals (Figure 8A). Values for IC50 were on average 0.25 mM for *eat-3(ad426)* animals and 0.44 mM for wildtype (N2) animals (averages of four independent experiments). Increased sensitivity to paraquat is also observed with *eat-3(tm1107)* animals (Figure 8B), confirming that this effect is caused by loss of *eat-3* function. The sensitivity of *eat-3(ad426)* animals to paraquat is suppressed by the *drp-1* mutations in *eat-3(ad426); drp-1(cq5)* and in *eat-3(ad426); drp-1(cq11)* animals (Figure 8A–8B). These two *drp-1* mutations were isolated independently, confirming that they are the cause of this reversal. Since these results suggests that mitochondrial outer membrane fission and fusion processes affect paraquat sensitivity, we tested whether the *fzo-1(tm1133)* mutant, which has a defect in mitochondrial outer membrane fusion, are also sensitive to paraquat. Our results show that this mutant is not more sensitive to paraquat than wildtype animals (Figure 8A), from which we conclude that mitochondrial fusion defects are not enough to promote free radical damage. The increased paraquat sensitivity of *eat-3* mutants, but not of *fzo-1* mutants, therefore indicates that the EAT-3 protein affects free radical formation or sequestration in ways that are unrelated to its role in mitochondrial fusion.

### Enhancement of the eat-3 Mutant Phenotype by SOD-2 Loss of Function

To test whether the induction of superoxide dismutase genes aids survival of *eat-3* mutants, we tested possible genetic interactions between *eat-3* and superoxide dismutase genes in *C. elegans*. *C. elegans* has five superoxide dismutase genes. The *sod-1, sod-4,* and *sod-5* genes encode Cu2+/Zn2+ superoxide dismutases. One splice variant of *sod-1* and all variants of *sod-4* have a signal peptide, suggesting that these proteins are sent through the secretory pathway to the extracellular matrix. Other *sod-1* isoforms and all proteins encoded by *sod-5* lack recognizable targeting sequences, suggesting that those are cytosolic. A fraction of Cu2+/Zn2+ superoxide dismutases might also be localized to the mitochondrial intermembrane even without recognizable targeting sequences, similar to Cu2+/Zn2+ superoxide dismutases in yeast and mammals [58]. The two remaining sod genes (*sod-2* and *sod-3*) encode Fe/Mn superoxide dismutases. These proteins have mitochondrial leader sequences, which most likely target them to the mitochondrial matrix.

**Figure 9:**
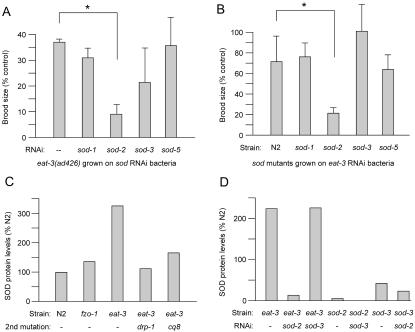
Increased Sensitivity to the Mitochondrial Matrix Superoxide Dismutase *sod-2.* (A) Effects of *sod-1, sod-2, sod-3,* and *sod-5* RNAi on the broodsize of *eat-3(ad426)* animals. The histogram shows the numbers of hatched worms on plates with *eat-3(ad426)* animals with or without *sod* feeding RNAi as percentages of the numbers of hatched worms on plates with wildtype (N2) worms with or without feeding RNAi. (B) Effects of *eat-3* RNAi on the broodsizes of *sod-1, sod-2, sod-3* and *sod-5* mutants. Wildtype (N2) worms are compared with the *sod* mutants, *sod-1(tm776)*, *sod-2(gk257)*, *sod-3(gk235), sod-4(gk101),* and *sod-5(tm1146).* The histogram shows the numbers of hatched worms on plates with *eat-3* feeding RNAi as percentages of the numbers of hatched worms on plates without feeding RNAi (vector alone). Both (A) and (B) show the average results for three independent experiments. For each point in each experiment five L4 larvae were transferred to individual bacterial plates with or without feeding RNAi. Eggs that hatched on those plates were counted as viable progeny. In each experiment the average values of five plates were determined. The error bars show SD for variation between the three experiments. An unpaired Student's *t* test was used for statistical analysis. The asterisk indicates P<0.005 in (A) and P<0.05 in (B) (n = 3). (C) Expression levels of Fe/Mn-SOD proteins relative to the expression levels in wildtype (N2) animals. Expression was determined by probing Western blots with an antibody that detects SOD-2 and SOD-3 proteins. Densitometric scans of SOD were normalized to tubulin levels for each lane and then expressed as a percentage of wildtype (N2) levels. The mutant strains used here have *fzo-1(tm1133)*, *eat-3(ad426)*, *eat-3(ad426); drp-1(cq5)* and *eat-3(ad426cq8)* alleles. (D) Expression levels in mutant animals grown with or without feeding RNAi bacteria, as indicated below the histogram. The mutant alleles used here are *eat-3(ad426)*, *sod-2(gk257)* and *sod-3(gk235)*.

We first grew *eat-3(ad426)* animals on feeding RNAi bacteria with RNAs for the *sod* genes that are not secreted (*sod-1*, *sod-2*, *sod-3* and *sod–5*), since those might affect the survival of *eat-3* mutants. There were little or at best modest effects with *sod-1*, *sod-3* and *sod-5* RNAi treatments, but the effects of *sod-2* RNAi on *eat-3(ad426)* animals were consistent and strong (Figure 9A). To verify these differences, we grew mutants of each of the sod genes on *eat-3* RNAi bacteria. As with the converse experiment, *sod-2(gk257)* mutant animals grow much more poorly with *eat-3* RNAi (Figure 9B). The effectiveness of *eat-3(ad426)* in one experiment and *eat-3* RNAi in the second experiment confirms that the enhancement of *sod-2* defects are indeed caused by *eat-3* loss of function. We conclude *sod-1*, *sod-3* and *sod-5* are not necessary for survival of the *eat-3* mutant, but a mutation in the *sod-2* gene and *sod-2* RNAi both strongly affect survival of animals with *eat-3* deficiencies.

The weak or negligible enhancement of *eat-3* by *sod-3* RNAi and the *sod-3(gk235)* mutant is noteworthy since SOD-2 and SOD-3 have 88% amino acid identity and both proteins have mitochondrial leader sequences, indicating that they are both targeted to the mitochondrial matrix. The genetic interactions between *sod-2* and *eat-3* might, however, be different from those between *sod-3* and *eat-3*, because *sod-2* and *sod-3* genes are differentially expressed [59] and their expression is regulated by different pathways [60]. We used Western blots probed with a cross reacting Fe/Mn-SOD antibody to determine whether differential expression of *sod* genes correlates with the different effects that we observe with *sod-2* and *sod-3* genes.

Our blots show that Fe/Mn-SOD expression is induced more than two-fold in *eat-3(ad426)* animals (Figure 9C). This induction is almost entirely attributable to SOD-2 since *sod-2* RNAi, but not *sod-3* RNAi largely abolishes this expression (Figure 9D). The induction is reversed by a secondary mutation in *drp-1(cq5)* and in the intragenic revertant of *eat-3(ad426cq8)* (Figure 9C). Similar reductions were seen with other revertants (data not shown). Consistent with their lack of paraquat sensitivity, *fzo-1(tm1133)* animals show little or no induction of SOD expression. We conclude that SOD-2 protein levels are dramatically increased in *eat-3(ad426)* animals, but not in *fzo-1(tm1133)* animals. This increase is partially reversed in intragenic revertants and fully reversed by the *drp-1* mutation in the *eat-3(ad426); drp-1(cq5)* double mutant. It seems likely that increased expression of SOD-2 helps prevent damage from free radicals, but this increase is still not enough to prevent the hypersensitivity of *eat-3* mutants to paraquat.

## Discussion

*C. elegans eat-3* mutants have many of the same features that were previously observed with yeast Mgm1 mutants and mammalian cells transfected with Opa1 siRNA. The mitochondria in *eat-3* mutants are fragmented, these fragmented mitochondria are further divided by inner membrane septae and fragmentation is reversed by loss of Drp1. *C. elegans eat-3* mutants are also affected at the organismal level. The mutant animals grow slowly, are sluggish and have greatly reduced broodsize, consistent with severely compromised mitochondrial function. However, heterozygous *eat-3* mutations have no overt defects in worms, unlike heterozygous Opa1 mutations in humans, which cause optic neuropathies through haploinsufficiency. The *eat-3* mutants are nevertheless still useful for unraveling pathogenic mechanisms, since the phenotypes in *C. elegans* and in mammal are both due to loss of protein function and therefore their effects on other cellular pathways are also most likely similar.

It was conceivable that the broodsize defects of *eat-3* mutants are due to increased apoptosis in the gonad. In wildtype worms, approximately 50% of germ cells die prior to oogenesis, but more death can be induced by DNA damage, by pathogens and by other forms of stress. These death-inducing conditions all converge on the classic apoptosis machinery that requires the caspase CED-3 and the APAF1 homologue CED-4 [61]. We investigated the possibility that apoptosis contributes to the pathogenesis of *eat-3* mutants by analyzing *eat-3*; *ced-3* and *eat-3*; *ced-4* double mutants and by counting the numbers of dying cells in *eat-3* mutants. There was no increase in the numbers of dying cells in *eat-3* embryos, nor was there suppression of the *eat-3* broodsize defects in the double mutants. *C. elegans* does have several other caspases (*csp-1*, *csp-2* and *csp-3*), but RNAi of these genes had no effect on *eat-3* animals (data not shown) nor are they known to contribute to apoptotic cell death in *C. elegan*s [56]. Redundancy is not an issue with *ced-4*, which encodes the only APAF1 homologue in *C. elegans*. In summary, none of the RNAi treatments or chromosomal mutations in cell death genes showed a suppressive effect on *eat-3* mutants, from which we conclude that caspase-dependent cell death does not contribute to the pathology of *eat-3* in worms.

Mammalian cells transfected with Opa1 siRNA are more sensitive to apoptosis inducing agents [62], but there is also evidence that patients with dominant optic atrophy have reduced levels of ATP, which could trigger retinal ganglion cell degeneration [42]. The gross anatomical phenotypes of *C. elegans eat-3* mutants, such as small size, slow growth and reduced broodsize are consistent with caloric restriction as observed in feeding mutants with pharyngeal defects [43],[52]. The small brood sizes of *eat-3* mutants are not due to retention of eggs, nor are there increased numbers of dead eggs or larvae on plates (data not shown). There is, however, a paucity of nuclei in the gonads of eat-3 RNAi animals (Figure 5C), consistent with the production of fewer oocytes. Fewer oocytes could reflect reduced rates of mitotic division at the distal tip of the gonad, since it was previously shown that mutations in mitochondrial proteins can inhibit cell division through the actions of AMP kinase and cyclin E [63]. Oocyte production might also be compromised at later stages, since the availability of yolk protein and other major constituents of oocytes is affected by the metabolic state of the animal. Many of the *eat-3* mutant phenotypes are therefore attributable to a general breakdown in mitochondrial function.

Earlier studies of yeast Mgm1 mutants show progressive loss of mtDNA [18],[19]. Loss of mtDNA is also observed in patients with dominant optic atrophy where it will affect assembly of oxidative phosphorylation complexes [64]. Defects in oxidative phosphorylation proteins can result in fewer and shorter cristae [65], which would be confined to those matrix compartments that have lost their mtDNA. Selective loss of cristae due to stochastic loss of mtDNA agrees with our electron microscopy data, since that data shows a heterogeneous mixture of mitochondrial matrix compartments, some with severely disrupted cristae and others with seemingly wildtype cristae (pairs of arrows in Figure 3D). The observation of different types of matrices enclosed by a single mitochondrial outer membrane suggests that the outer membranes of *eat-3* mutant mitochondria fuse irrespective of their mtDNA content, but the mitochondrial inner membranes fail to fuse, similar to the results obtained with Mgm1 in yeast [25].

The ability to suppress *eat-3(ad426)* defects with *drp-1* RNAi and mutations in *drp-1* is consistent with stochastic loss of mtDNA in *eat-3* mutants. Mutations in the yeast DRP-1 homologue Dnm1 similarly suppress Mgm1 growth defects and they restore cristae morphology [20]. There are, however, several observations suggesting that the mitochondrial fusion defect and the resulting loss of mtDNA might not be the only causes of sickness in *eat-3* mutants: First, *drp-1* RNAi does not rescue the *C. elegans eat-3(tm1107)* deletion allele, while it does rescue the *eat-3(ad426)* allele. Second, *C. elegans fzo-1(tm1133)* mutant animals are not as severely affected as *eat-3* mutants, nor are they rescued by *drp-1* RNAi (data not shown), even though one might expect them to be equally susceptible to loss of mtDNA, since yeast Fzo1 mutants do lose their mtDNA [12],[48] and they are rescued by mutations in Dnm1 [19],[21],[22]. Extensive loss of mtDNA also occurs in mouse Mitofusin mutants (the mammalian homologues of Fzo1) [66]. We conclude that lack of ATP due to loss of mtDNA is not enough to explain why optic nerves are singled out for destruction in patients with dominant optic atrophy.

Our results suggest an alternative explanation for the sickness of *C. elegans eat-3* mutants, which may also be relevant for the selective degeneration of retinal ganglion cells in patients with dominant optic atrophy. *C. elegans eat-3* mutants are hypersensitive to paraquat and *sod-2* RNAi, suggesting increased production of free radicals or an impaired disposal mechanism. A *drp-1; eat-3* double mutant and an *fzo-1* mutant are not more sensitive to paraquat, suggesting that there might be something specific about the effects of *eat-3* on mitochondria, for example contributing to the maintenance of cristae, as was suggested for Opa1 in mammalian cells [27],[37],[40],[55],[67]. The enhancement of *eat-3* phenotypes by *sod-2* RNAi and a mutation in the *sod-2* gene, but not by RNAi or mutations in other superoxide dismutase genes, suggests that damage from free radicals is confined to the mitochondrial matrix or the mitochondrial inner membrane. The effects are most likely not direct, since SOD-2 is a mitochondrial matrix protein while EAT-3 is primarily localized to the mitochondrial intermembrane space and other mutations that affect oxidative phosphorylation in *C. elegans*, such as the *mev-1* and *gas-1* mutants with mutations in complex I and II proteins, also show increased sensitivity to paraquat [57]. Disruption of the electron transport chain, for example through altered cristae morphology, can increase production of free radicals, while conversely free radicals in the mitochondrial matrix can further disrupt the electron transport chain. These two problems are therefore likely to reinforce each other, possibly leading to catastrophic breakdown of mitochondrial function.

If free radicals also contribute to dominant optic atrophy in humans, then the underlying cause of this disease might be more similar to that of other optic neuropathies than previously understood. Patients with Leber's hereditary neuropathy (LHON) have mutations in subunits of Oxidative Phosphorylation complex I, which increases free radical production by disrupting the flow of electrons through complex I along with their more obvious effects on ATP production [68]-[70]. Optic neuropathies triggered by macular degeneration and optic neuropathies triggered by dietary deficiencies are also linked to damage from free radicals in mitochondria. Increased levels of free radicals in these diseases are compounded by the effects of light entering the eyes, since light triggers additional free radical production through absorption by cytochrome c oxidase and flavin containing oxidases in mitochondria [71]. Damage from free radicals will exacerbate the effects of ATP deficiency and increased susceptibility to apoptosis in patients with dominant optic atrophy. It is even possible that some of the increased susceptibility to apoptosis in Opa1 deficient cells is caused by damage from free radicals.

In conclusion, mutations in *C. elegans eat-3* have many of the same effects on mitochondrial morphology that were previously observed with mutations in yeast Mgm1 and mammalian Opa1. Mutations in key components of the major cell death pathway show that this pathway does not affect the *eat-3* phenotype. Instead, *eat-3* mutants are sensitive to damage from free radicals and they show hallmarks of ATP deficiency. The effects of *sod-2* loss of function and partial compensation by induced expression of SOD-2 suggest that damage from free radicals is localized to the mitochondrial matrix. These observations might help design more effective treatments for patients with DOA.

## Materials and Methods

### Molecular Cloning

The D2013.5 gene of *eat-3(ad426)* was sequenced using amplified genomic DNA from two independent PCR reactions. The *C. elegans eat-3* cDNAs yk10h8 and yk21c2 were obtained from Y. Kohara (National Institute of Genetics, Mishima, Japan). The pPD expression vectors were kindly provided by A. Fire, J. Ahnn, G. Seydoux, and S. Xu (Carnegie Institution of Washington, Baltimore, Maryland). The P*eat-3::NLS::GFP::β-galactosidase* construct was made with an *eat-3* gene promoter fragment (positions 23335 to 25288 of cosmid D2013), fused to the reporter sequences of pPD95.67. The rescue construct contained this same promoter fragment fused to the yk21c2 cDNA. This cDNA lacks the N-terminal 70 amino acids. The missing sequence was generated by PCR of genomic DNA. Mutations were introduced by PCR and verified by sequencing**.** EAT-3 was expressed in muscle cells using the *myo-3* promoter of pPD96.52. The antisense construct has the insert of yk21c2 cloned in the antisense orientation in pPD96.52. Production of dsRNA, mitochondrial markers, microinjection, light microscopy and feeding RNAi procedures were described previously [8],[72]. Feeding RNAi bacteria were kindly provided by Dr. J. Ahringer (University of Cambridge, UK).

### Worm Strains

*C. elegans* strains were obtained from the *C. elegans* stock-center (CGC, University of Minnesota) and from Dr. S. Mitani (National Bioresource Project of Japan. Tokyo Women's Medical University School of Medicine, Tokyo). Strains provided by Dr. Mitani were backcrossed with wildtype (N2) animals to remove adventitious mutations. Revertants of *dyn-1(ky51)* and *eat-3(ad426)* were generated with EMS mutagenesis. The *dyn-1(ky51)* is temperature sensitive for growth and motility [46]. L3 larvae of either strain were treated with 50 mM EMS as described [73]. F2 progeny of mutagenized animals were screened for revertants by looking for restored growth and motility. The *dyn-1(ky51)* animals were screened at the restrictive temperature (25°C) while *eat-3(ad426)* animals were screened at 20°C. Newly identified mutants were backcrossed with wildtype (N2) worms to determine whether the new mutations are intra- or extragenic and to rid them of adventitious mutations. The three revertants of *dyn-1(ky51)* were genetically inseparable from the original *dyn-1* mutation and five of the seven *eat-3(ad426)* revertants were inseparable from *eat-3*, suggesting that these are intragenic revertants. New mutations in the intragenic revertants were identified by sequencing their respective *dyn-1* and *eat-3* genes. New mutations in the two extragenic revertants of *eat-3(ad426)* were identified by sequencing their *drp-1* genes.

To determine paraquat sensitivity, increasing concentrations of paraquat (N,N′-Dimethyl-4,4′-bipyridinium dichloride from MP Biomedicals LLC, Solon Ohio) were added to 30 mm NGM agar plates. These plates were seeded with OP50 bacteria [73] and fifty L1 larvae were transferred to each plate. The plates with worms were incubated at 20°C and tracked for several days by counting the numbers of worms that reached adulthood.

### Electron Microscopy

Young gravid worms were mixed with *E. coli* or dry baker's yeast and 10% methanol [74]. This mixture was cryofixed in a Bal-Tec HPM 010 high pressure freezer (Technotrade, Manchester, New Hampshire), followed by freeze-substitution with 2% osmium tetroxide and 0.1% uranyl acetate in acetone. The temperature was slowly increased to −20°C and then to room temperature. The samples were rinsed with acetone and infiltrated with Epon-Araldite (1 hr in 1 part resin and 3 parts acetone; 2 hr in a 1∶1 mixture; 4 hr in a 3∶1 mixture; 1 hr and 16 hr in resin alone). The samples were then incubated in resin with accelerator for 4 hr, flat-embedded between Teflon-coated slides and cured in a 60°C oven for 48 hr. Longitudinal sections (60 nm thick) were post-stained with uranyl acetate and lead citrate. All specimens were examined using a Tecnai 12 transmission electron microscope at 100 kV. Membrane lengths and surface areas were measured with NIH Image software.

For tomography, 500 nm thick sections were cut and stained with uranyl-acetate and lead citrate. Colloidal gold particles (10 nm) were applied as alignment markers. A tilt series of 122 images was made on the Albany AEI EM7 MkII HVEM at 1000 kV. The images were recorded around two orthogonal tilt axes, each over an angular range of 120° with a 2° tilt interval. The double-tilt images were aligned, further processed to make a tomographic reconstruction, followed by surface rendering as previously described [75].

### Western Blotting

Samples for Western blot analysis were prepared by freeze/thawing worms, followed by solubulization in SDS-PAGE sample buffer, boiling for 10 min and clearing of debris by centrifugation for 2 min at 3,000 rpm in an Eppendorf microfuge. Western blots were probed with superoxide dismutase antibody from Abcam (Cambridge, Massachusetts). Western blots were quantified with densitometry using a Personal Densitometer SI and ImageQuant software (Molecular Dynamics, Sunnyvale, California).

## Supporting Information

Figure S1 Western blot showing EAT-3 expression levels in wild type and mutant *C. elegans*.
An antibody raised against recombinant *C. elegans* EAT-3 protein detects a strong band of approximately 90 kDa in all strains except for *eat-3(tm1107)*, which has a deletion in the *eat-3* gene. This band is the size predicted for mature protein, assuming multi-step processing similar to that of yeast Mgm1. A faint upper band of approximately 100 kDa is also detected in all strains except for *eat-3(tm1107)*. This upper band most likely results from the initial cleavage of the mitochondrial leader sequence (computer algorithms predict a product of 99 kDa). The line between lanes with *eat-3(ad426)*; *drp-1(cq5)* and *eat-3(tm1107)* samples shows that an empty lane between the two, which served as a buffer against spillover, was cut out. Tubulin and cytochrome serve as loading controls. The EAT-3 antibody was raised in a rabbit against recombinant protein. The recombinant protein was made by expression in bacteria with a his-tag and purified with Ni-NTA column chromatography. The serum was blot purified [76] and used for Western blotting as described in the Materials and Methods section. Tubulin antibody was from Sigma and cytochrome c antibody was from Pharmingen. Those were raised against mammalian proteins but show sufficient cross-reactivity with *C. elegans* proteins for Western blots.
(0.51 MB TIF)

Figure S2 Reversal of mitochondrial fragmentation in *eat-3(tm1107)* animals.
(A) Mitochondria in muscle cells of an *eat-3(tm1107)* animal stained with the membrane potential dependent dye Rhodamine 6G [8].
(B) Mitochondria in muscle cells of an *eat-3(tm1107)* animal grown with *drp-1* feeding RNAi showing reversal of the fragmented phenotype. This indicates that *drp-1* loss of function is epistatic to an *eat-3* null allele. The scale bar is 5 µm.
(1.72 MB TIF)
