# Supplementary figures and images for: The C. elegans Opa1 Homologue EAT-3 Is Essential for Resistance to Free Radicals
Source: PLoS Genet. 2008 Feb 29;4(2):e1000022. doi: 10.1371/journal.pgen.1000022 (PMC2265488; doi:10.1371/journal.pgen.1000022)

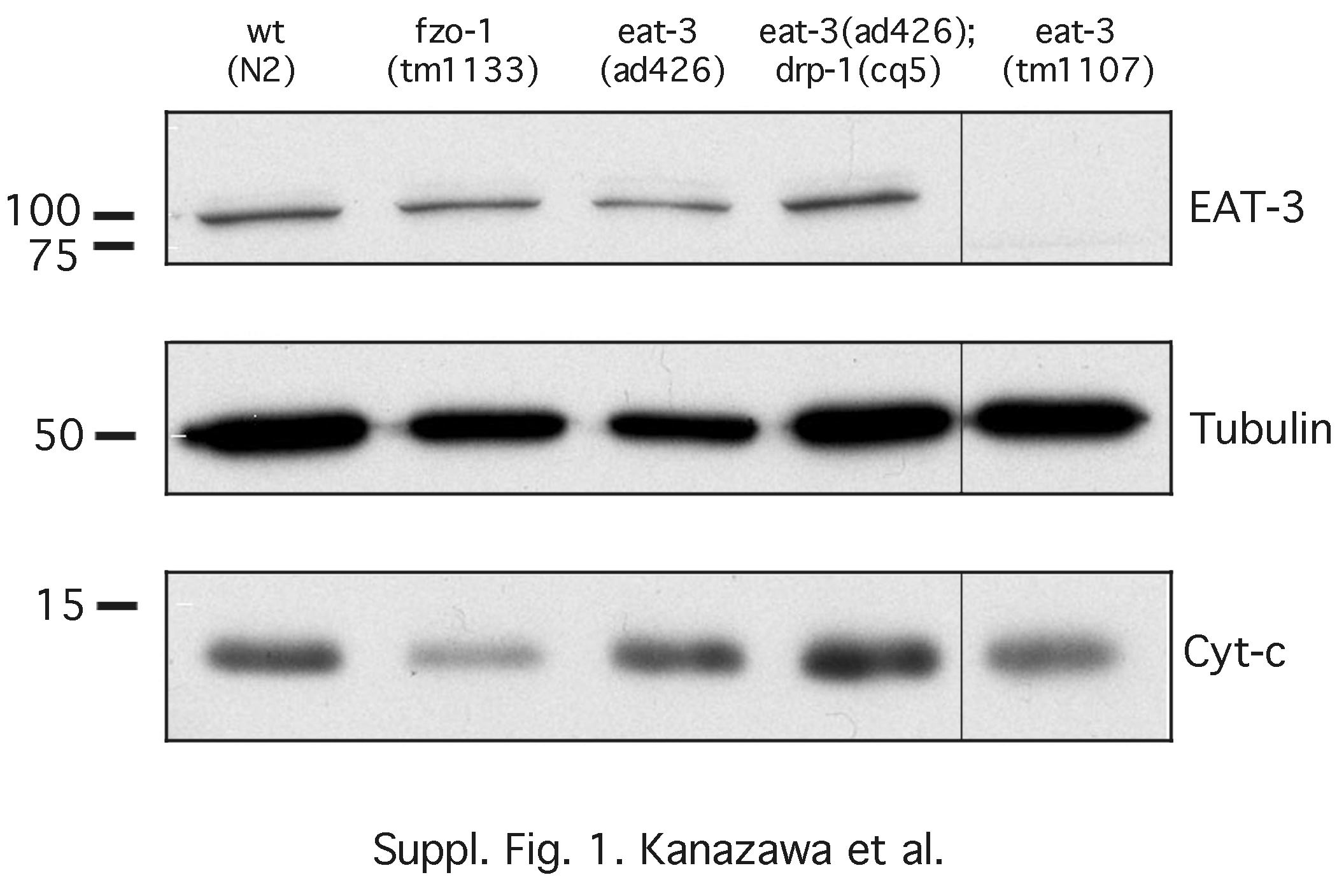

Supplement: Figure S1 — Western blot showing EAT-3 expression levels in wild type and mutant C. elegans. An antibody raised against recombinant C. elegans EAT-3 protein detects a strong band of approximately 90 kDa in all strains except for eat-3(tm1107), which has a deletion in the eat-3 gene. This band is the size predicted for mature protein, assuming multi-step processing similar to that of yeast Mgm1. A faint upper band of approximately 100 kDa is also detected in all strains except for eat-3(tm1107). This upper band most likely results from the initial cleavage of the mitochondrial leader sequence (computer algorithms predict a product of 99 kDa). The line between lanes with eat-3(ad426); drp-1(cq5) and eat-3(tm1107) samples shows that an empty lane between the two, which served as a buffer against spillover, was cut out. Tubulin and cytochrome serve as loading controls. The EAT-3 antibody was raised in a rabbit against recombinant protein. The recombinant protein was made by expression in bacteria with a his-tag and purified with Ni-NTA column chromatography. The serum was blot purified [76] and used for Western blotting as described in the Materials and Methods section. Tubulin antibody was from Sigma and cytochrome c antibody was from Pharmingen. Those were raised against mammalian proteins but show sufficient cross-reactivity with C. elegans proteins for Western blots. (0.51 MB TIF) [file pgen.1000022.s001.tif]

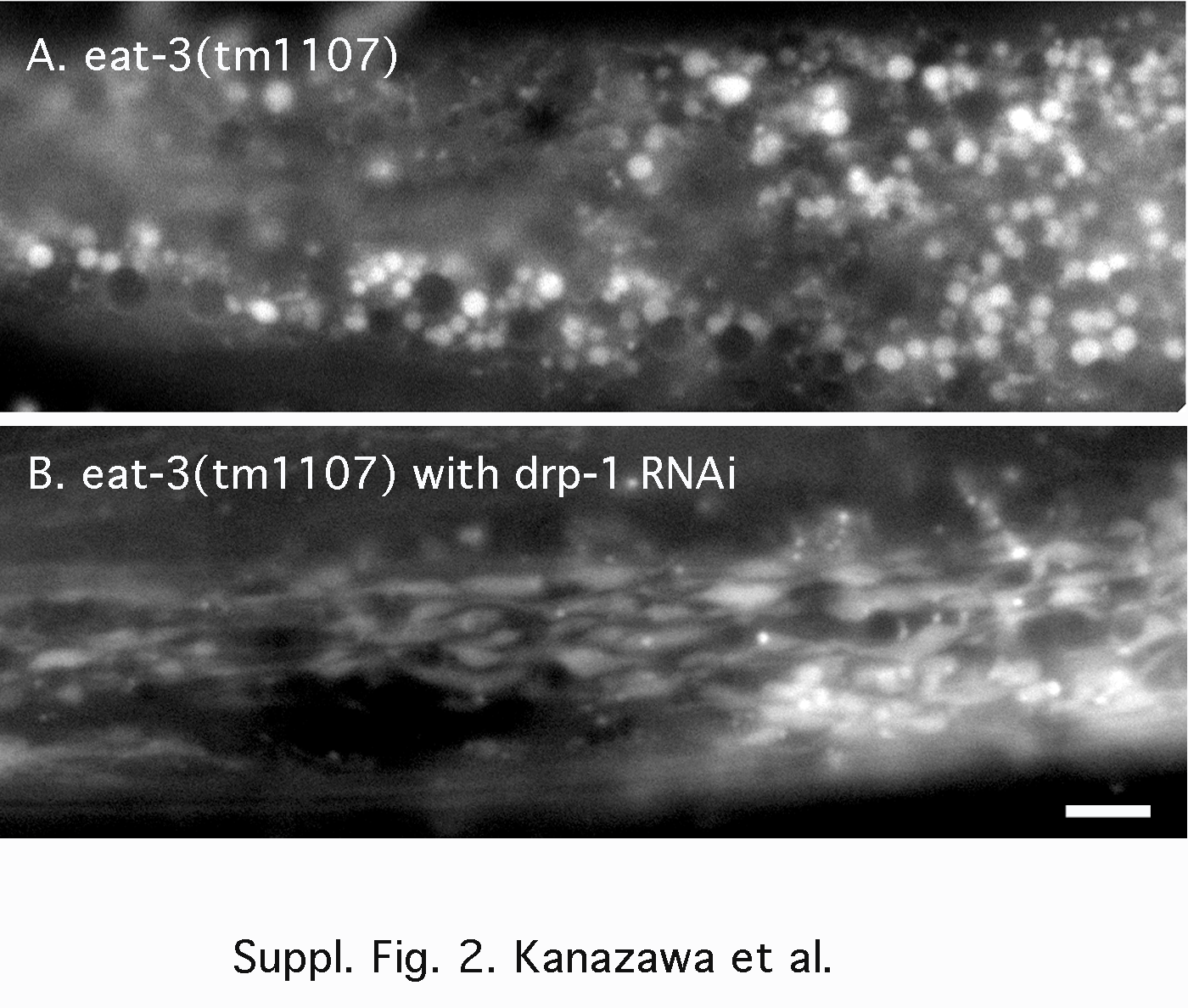

Supplement: Figure S2 — Reversal of mitochondrial fragmentation in eat-3(tm1107) animals. (A) Mitochondria in muscle cells of an eat-3(tm1107) animal stained with the membrane potential dependent dye Rhodamine 6G [8]. (B) Mitochondria in muscle cells of an eat-3(tm1107) animal grown with drp-1 feeding RNAi showing reversal of the fragmented phenotype. This indicates that drp-1 loss of function is epistatic to an eat-3 null allele. The scale bar is 5 µm. (1.72 MB TIF) [file pgen.1000022.s002.tif]
